# Identifying primary care datasets and perspectives on their secondary use: a survey of Australian data users and custodians

**DOI:** 10.1186/s12911-022-01830-9

**Published:** 2022-04-06

**Authors:** Rachel Canaway, Douglas Boyle, Jo-Anne Manski-Nankervis, Kathleen Gray

**Affiliations:** 1 Department of General Practice, Melbourne Medical School, Faculty of Medicine, Dentistry and Health Sciences, The University of Melbourne, Parkville, VIC 3010 Australia; 2 School of Computing and Information Systems and Melbourne Medical School, The University of Melbourne, Parkville, VIC 3010 Australia

**Keywords:** Australia, Data curation, Data linkage, Data management, Data quality, Primary health care data, Secondary use of data

## Abstract

**Background:**

Most people receive most of their health care in in Australia in primary care, yet researchers and policymakers have limited access to resulting clinical data. Widening access to primary care data and linking it with hospital or other data can contribute to research informing policy and provision of services and care; however, limitations of primary care data and barriers to access curtail its use. The Australian Health Research Alliance (AHRA) is seeking to build capacity in data-driven healthcare improvement; this study formed part of its workplan.

**Methods:**

The study aimed to build capacity for data driven healthcare improvement through identifying primary care datasets in Australia available for secondary use and understand data quality frameworks being applied to them, and factors affecting national capacity for secondary use of primary care data from the perspectives of data custodians and users. Purposive and snowball sampling were used to disseminate a questionnaire and respondents were invited to contribute additional information via semi-structured interviews.

**Results:**

Sixty-two respondents collectively named 106 datasets from eclectic sources, indicating a broad conceptualisation of what a primary care dataset available for secondary use is. The datasets were generated from multiple clinical software systems, using different data extraction tools, resulting in non-standardised data structures. Use of non-standard data quality frameworks were described by two-thirds of data custodians. Building trust between citizens, clinicians, third party data custodians and data end-users was considered by many to be a key enabler to improve primary care data quality and efficiencies related to secondary use. Trust building qualities included meaningful stakeholder engagement, transparency, strong leadership, shared vision, robust data security and data privacy protection. Resources to improve capacity for primary care data access and use were sought for data collection tool improvements, workforce upskilling and education, incentivising data collection and making data access more affordable.

**Conclusions:**

The large number of identified Australian primary care related datasets suggests duplication of labour related to data collection, preparation and utilisation. Benefits of secondary use of primary care data were many, and strong national leadership is required to reach consensus on how to address limitations and barriers, for example accreditation of EMR clinical software systems and the adoption of agreed data and quality standards at all stages of the clinical and research data-use lifecycle. The study informed the workplan of AHRA’s Transformational Data Collaboration to improve partner engagement and use of clinical data for research.

**Supplementary Information:**

The online version contains supplementary material available at 10.1186/s12911-022-01830-9.

## Background

Primary care is the most commonly visited health sector in Australia with 83% of the population visiting a general practitioner (GP) in a given year [1]. Yet despite most people receiving the majority of their health care in the primary and community care sector, limited secondary use is made of resulting clinical and administrative data [2]. Secondary use of data refers to its use for purposes other than those that they were originally collected including: research, quality assurance, surveillance, audit, and record linkage with other routinely collected data (e.g. from hospitals, other healthcare providers, education, social services). Australia’s secondary use of health data is reportedly behind many developed countries, this limits knowledge of patients’ journeys through the healthcare system [3]. Access to primary care data in Australia is a particular concern [4–6] and activities are underway to address this gap [2, 7, 8]. Internationally there is no consensus on good practice; the structures and extent of secondary use of primary care-related data are far from universal [9–15], and governance, privacy protection, data quality, completeness, bias, technical and value-based (consent) barriers to data use are acknowledged as needing to be addressed [12, 16–22].

The Australian general practice sector constitutes mainly private businesses that are partially or wholly funded by national government Medicare Benefit Scheme (MBS) rebates (government funding scheme for public healthcare services). By 2005 the sector was estimated to have achieved around 90% clinical computerisation [23, 24], well ahead of Australia’s hospital sector which is more recent in its implementation of electronic medical records (EMRs) [25]. GPs collect reason for visit, diagnoses, patient history, prescribing, pathology and diagnostic tests and results, consultation types and referrals. Some general practices voluntarily provide aggregated or individuals’ clinical encounter data to Primary Health Networks (PHNs)—government funded entities responsible for increasing efficiency and effectiveness of primary health services [26]) or to repositories which use the data for the secondary purposes of disease surveillance and monitoring and research for policy and population health planning purposes [2, 27, 28].

Data are collected in general practice using a range of commercial clinical software systems, data extraction occurs using a variety of data extraction tools, and extracted data are housed in a variety of repositories in different formats; there are no mechanisms for standardisation or accreditation of the data collection or extraction tools [2]. As is common in many countries, Australia lacks national governance and infrastructure mechanisms for primary and other health data housed in different repositories to be combined and made available to support research and healthcare planning.

Government support for clinical data sharing in Australia is emergent. The Australian Government’s ‘My Health Record’ became widely adopted in 2018 when it changed from opt-in to opt-out citizen registration. ‘My Health Record’ is an online summary of individual’s health information that can be contributed to by medical professionals and individuals [29]. It requires consent from patients for GP (or other care provider) upload of patient summary data. While there is a ‘Framework to guide the secondary use of My Health Record system data’ [30], the actual data are not yet available for secondary use [31]. In planning is a ‘National Primary Health Care Data Asset’ for which the Australian Institute for Health and Welfare (AIHW) commenced stakeholder consultation in 2019 [32]. In August 2019, the Australian government also introduced the ‘Quality Improvement Practice Incentive Payment (QI PIP)’ to incentivise general practices to share an ‘Eligible Data Set’ with their local Primary Health Network (PHN) and participate in a program of data-led “continuous quality improvement” [33]. The impact of this program has not yet been evaluated. Based on these arrangements, overall sharing of primary care data in Australia is voluntary and not necessarily representative. Consequently, there is no comprehensive primary care data repository in Australia [27, 28]. This differs from the UK and the Netherlands, for example, where primary care data has long been routinely collected from clinicians’ EMRs and used for secondary purposes [10, 11] and where governments are taking steps to prompt interoperable clinical data collection tools [34, 35].

Secondary use of primary care data, including linkage with other datasets, can improve policymakers’ and researchers’ understanding of patient ‘journeys’ through the healthcare system [22, 36]. Most ‘secondary use’ of clinical and administrative data from primary care occurs across separate projects and institutions which leads to lack of visibility on whom are using certain data, how data are collected, its granularity, and the benefits and barriers to its collection and utilisation [2, 9, 12]. Until a national picture is comprehended, it is likely that resources are being duplicated and/or inefficiently used which leads to unnecessary economic burden [3, 37]. Furthermore, although data quality assurance is essential if research using clinical data is to provide meaningful outcomes, it is not currently visible whether or what data quality frameworks are being applied when preparing data for secondary use [38]. In Australia, the environment surrounding primary care data utilisation for secondary purposes is uncharted.

This study was commissioned by the Melbourne Academic Centre for Health (https://machaustralia.org) to address the Australian Health Research Alliance (AHRA) national system level initiative to build capacity in data-driven healthcare improvement (https://ahra.org.au/our-work/data-driven-healthcare-improvement). AHRA comprises seven centres of Advanced Health Research Translation and Innovation in Regional Health. In March 2018, subsequent to circulation of a modified Delphi survey, a workshop to define the data driven healthcare improvement priorities was held at the which problem definition and priority setting activities were undertaken [39]. The following fundamental problems framed the resulting workplan: “the unknown scope of data linkage activities utilising primary care data across Australia, and the distrust in the quality and completeness of routinely collected data in primary care across Australia”. The study below is a component of a larger workplan that included components on developing trust in routinely collected general practice data through advancing use of a data quality assessment framework [38], and a national stakeholder workshop (primary care data custodians and users) that explored preliminary data from this study and priority areas for improving primary care data quality and use. Reporting on the additional components is beyond the scope of this article. A non-peer reviewed report of the study was published [37] and here we provide a peer reviewed summary of the methods, findings and more nuanced discussion.

## Methods

### Aim

This component of the study aimed to gain a deeper understanding of the availability and secondary use of routinely collected primary care data (primarily general practice data) across Australia and in doing so, to contribute to building capacity for data-driven healthcare improvement [37].

### Study design

This exploratory study aligned with the Health Policy and Systems Research (HPSR) approach which enables research questions and strategy to be developed in a real-world, feasible manner in response to problems, which in this instance had been pre-defined by AHRA (see above). HPSR is a problem rather than method driven approach, drawing strongly on social science perspectives and seeking to support the development and implementation of applied policy and health system change [40, 41]. Members of the research team have experience in collection, curation and governance of sensitive health data and providing consultancy/expert advice on healthcare data use and linkage in Australia (see Declarations) and RC who undertook data collection and the majority of data analysis is an experienced health social scientist and policy researcher. These experiences have shaped interpretation of the findings.

This component of the study was designed to canvas expert stakeholder knowledge and perspectives to meet the study aim and answer the following key questions: What primary care datasets are available in Australia and are they being linked to other datasets? What (data) quality frameworks are being used to assess primary care datasets? What enablers and barriers should be considered to maximise the effectiveness of strategies to build benefit and capacity of primary care data? [37]. A secondary question, linked to the aim of building capacity for data-driven healthcare improvement, was: What are the perceived benefits and limitations of secondary use of primary care data?

To identify primary care related datasets for secondary use, and understand the strengths and weaknesses of the secondary use of primary healthcare data landscape from the perspectives of those engaging with it (data users or dataset custodians), we designed an online questionnaire for broad dissemination. Survey respondents were invited to contribute further information via phone to enable expansion of responses given in surveys and the exploration of additional themes deemed relevant by participants. A semi-structured interview guide and consent form were forwarded to responders who self-nominated for interview (see Additional files: 1 and 2). Although the main area of interest was general practice data we took a broad approach and sought information about ‘primary care data’.

In keeping with the HPSR approach, a workshop was held where preliminary findings were fed back to, and additional data collection undertaken with, stakeholders with an interest in the secondary use of general practice data. Reporting on the workshop is beyond the scope of this article [37]. Ethics approval and informed consent were obtained for each component of the study (see Declarations).

### Participant eligibility

Eligibility of survey responders was checked at commencement of the survey; first, secondary use of electronic medical records was defined, then the respondent was asked if they had used or had responsibility for a primary care dataset used for secondary purposes. ‘No’ responders exited the survey at this point. Those who responded ‘Yes’ were then asked whether they considered themselves a custodian or ‘owner’ of a general practice or other primary care dataset. Data owners branched to questions for data custodians and the others to questions for secondary ‘users’ of general practice or other primary care datasets. All responders were given the same ‘Building capacity of primary care datasets’ and ‘About you’ demographic questions (see Additional file: 1). No incentives were offered for participation, but participants could opt to be acknowledged in publications arising from the study.

### Data collection instrument

The online survey was created in REDCap (Research Electronic Data Capture tool [42]), hosted at the University of Melbourne. The survey included qualitative (long answer) and quantitative (demographic and closed ended) questions and was progressively piloted by eight stakeholders and iteratively modified to improve functionality, responder comprehension and analytical utility before it was distributed. The survey collected information about primary care-related datasets, application of quality frameworks, data linkage activities, and perspectives on limitations, benefits, barriers and enablers of secondary use of general practice data (see Additional file: 1).

### Data collection

Purposive and snowball sampling were used to disseminate the questionnaire broadly. The initial list of recipients was generated by the researchers based on their knowledge individuals and organisations that did or might have had an interest in secondary use of primary care data. Snowball sampling was used to encourage initial survey recipients to circulate the information and survey link among their team or to any other known primary care data users or custodians. We initially emailed the survey to 208 potential respondents. Invitees included members of the MACH Data Driven Healthcare Improvement Committee; known custodians of primary care data; academics in general practice/primary care-related academic departments, research institutes and research translation centres; government health departments and agencies including PHNs; primary care-related professional colleges, peak bodies and networks; Aboriginal controlled health organisations; health informatics networks and societies; general practice clinical software and data extraction companies; health insurer research bodies; and relevant consumer representative organisations. The e-mail and two reminders were sent during the survey open dates: 2 October to 9 November 2018.

Thirteen survey respondents volunteered for interview and seven formally consented. Interviews were undertaken via telephone in November 2018 (interview length ranged from 24 to 55 min, average 42 min), they were audio-recorded, transcribed and participants were able to review their transcript, with the exception that verbatim transcription of one audio recording of poor quality was not possible.

### Data analysis

Survey responses were checked for completeness in Excel, then imported into QSR NVivo 12 Plus [43] and SPSS Statistics version 26 [44]. In NVivo, the free text long and short answer responses were grouped by question, with responses to a question thematically coded or grouped into themes. The coding was an iterative process with themes emerging from and contained to each question/response group [45]. Within NVivo, categorically named primary care datasets were coded twice to ensure list accuracy, with the number of different respondents to name a particular dataset noted. SPSS Statistics was used to generate descriptive frequency and contingency tables for responses to the 17 categorical questions. Analyses of the interview transcripts, also facilitated by NVivo, occurred after analysis of the survey data and aimed to provide illustrative examples of issues arising. In keeping with the analysis, the results are ordered by survey question, preserving respondents’ interpretation of and perspectives on what are primary care datasets and issues relating to them. For example, Tables 5 and 6 outline the themes arising from the survey questions on benefits, limitations, barriers and enablers of secondary use of primary care data, and summarise respondents’ responses that gave rise to the themes. The results and discussion summarise and build from those provided in the report to funder [37].

## Results

### Participant characteristics

Of the 137 survey attempts recorded, 62 were eligible and provided sufficient data, 17 respondents marked themselves ineligible and so exited at the eligibility question: “*Have you used, or do you have responsibility for, a primary care dataset that is used for secondary purposes?”* [37], and 58 respondents provided insufficient or no data.

Of the 62 eligible questionnaires received, 32 (51.6%) were ‘Data Users’ and 30 (48.4%) ‘Data Custodians’. Table 1 summarises the jurisdiction of respondents and their organisational affiliations; the variation in respondent numbers between jurisdictions reflects the differing population sizes of the jurisdiction. Participants from educational/research institutions and PHNs made up 77.4% of the sample. Six researchers were also practising GPs.

**Table 1:** Jurisdiction of respondent affiliations, N = 62

Organisation	Total n (%)	Data custodians n (%)	Data users n (%)
Educational or Research Institute * (including universities)	24 (38.7)	8 (26.7)	16 (50.0)
Primary Health Network	24 (38.7)	16 (53.3)	8 (25.0)
Government	6 (9.7)	1 (3.3)	5 (15.6)
General Practice*	3 (4.8)	2 (6.7)	1 (3.1)
Pharmaceutical	1 (1.6)	Nil	1 (3.1)
Health insurer	Nil	Nil	Nil
Other (incl. software developer, non-Government/non-University data holder)	3 (4.8)	3 (10.0)	Nil
Not stated	1 (1.6)	Nil	1 (3.1)
State or Territory			
Australian Capital Territory	7 (11)	3	4
New South Wales	18 (29)	10	8
Northern Territory	3 (5)	0	3
Queensland	8 (13)	5	3
South Australia	3 (5)	1	2
Tasmania	2 (3)	0	2
Victoria	17 (27)	9	8
Western Australia	2 (3)	1	1
Elsewhere (National)	1 (2)	1	0
Not stated	1 (2)	0	1
Total	62 (100)	30 (100)	32 (100)

Table adapted from [37]

*Six of the persons from research/education institutions also worked in general practice. The 3 GPs noted worked only in general practice

The majority of respondents (n = 44, responses missing from n = 9) reported accessing, for secondary purposes, 1 to 5 primary care datasets; 6 reported accessing 11 + . Among data custodians, 3 reported not having accessed any.

Interviewees were data custodians (n = 4) and data users (n = 3) from New South Wales, Queensland, Tasmania and Victoria, representing four universities, two Primary Health Networks (PHNs), and a government health department [37].

### Primary care datasets

**Survey questions: (Custodians) ‘*What is the name of the dataset you are Custodian of or have responsibility for, and/or where it is located?’* (Users) ‘*What primary care or general practice datasets have you used for secondary purposes?’* (All) ‘*If you are aware of any other primary care datasets being used for secondary purposes, please list’.*** Participants collectively named 106 datasets that they associated with secondary use of primary care data. Datasets included those derived from EMRs, government collected administrative data (including MBS and Pharmaceutical Benefits Scheme [PBS] data), bespoke research collections, and collections from other community healthcare service providers. While most datasets contained patient-level data that related directly to care or research (mostly deidentified), some respondents identified datasets concerning health workforce, registries (e.g. immunisation register), sentinel practices (used for population health surveillance) and pathology results. Named datasets were held by government departments and government agencies, universities and research institutes, primary care providers such as GPs and community services and community mental health agencies, Aboriginal Medical Services, pharmacy related data, alcohol and other drug agencies, vendors of GP clinical software systems, and others. The most commonly named datasets (referred to by 3 or more participants) are listed in Table 2. The full list of named datasets is available elsewhere [37].

**Table 2:** Most commonly identified primary care datasets for ‘secondary use’ and their characteristics identified by respondents

Times mentioned	Dataset used for secondary purposes	Jurisdiction	Data extraction/collection tool	Purpose
> 20	Primary Health Network (PHN) collected data (individual datasets held by 28 PHNs)	National	PenCS tools, POLAR, Primary Sense	Audit, health planning, quality improvement, sometimes research
20	NPS MedicineInsight	National	GRHANITE® and cdmNET	Post market surveillance, audit, research
11	BEACH (Bettering the Evaluation and Care of Health) data (1998–2016)	National	Paper-based data collection	Research
11	Outcome Health and POLAR data	NSW, Victoria	POLAR	Audit (used by PHNs), research
11	Medical Benefits Scheme (MBS) data	National	Administrative claims	Administrative, audit, research
11	Pharmaceutical Benefits Scheme (PBS) data	National	Administrative claims	Administrative, audit, research
9	PHN related Primary Mental Health Care Minimum Data Set	National	PenCS tools	Audit, health planning, quality improvement,
7	Patron primary care data repository/Data for Decisions (University of Melbourne)	Victoria	GRHANITE®	Research
4	Aboriginal Community Controlled Organisations/Aboriginal Medical Services	National	Administrative data	Clinical care, audit
4	Australian Institute of Health and Welfare (AIHW) held data (**in addition** AIHW was mentioned in relation to access to other AIHW held datasets such as PBS, MBS)	National	Administrative data	Audit, health planning
3	Australian Immunisation Register (AIR)	National	Administrative data	Audit, surveillance
3	University of NSW ePractice-Based Research Network data	NSW	GRHANITE®	Research
3	Medical Director (clinical software vendor held data)	National	Cloud-based collection	Not stated
3	10% MBS and PBS sample data (no longer available)	National	Administrative claims	Research
3	Patient Reported Experience Measures (Australian Bureau of Statistics)	National	Household survey questionnaire	Care planning
3	My Health Record	National	Cloud-based collection	Clinical care, research

Table adapted from [37]

NSW, New South Wales

**Survey questions: (Custodians) ‘*Please describe characteristics and purpose of the dataset(s)’;* (Users) ‘*Please describe the nature of your interaction with these datasets’.*** Dataset purpose is summarised in Table 2. Datasets held by research institutions, NPS MedicineWise, and some government or government agencies were used for research including clinical trials and many had audit functions. Some PHN respondents suggested their primary care data were used for research, but such ‘research’ was generally described as internal analysis undertaken for population health planning and needs assessments. PHN datasets were used extensively for population health and service planning, disease surveillance, audit, post-market surveillance of medicines, and to identify opportunities for quality improvement.

The names of data extraction tools used were not explicitly asked for, but in describing the datasets, the following tools for extracting data from general practices were named: PenCS tools (widely used by PHNs), POLAR (Outcome Health), GRHANITE (University of Melbourne), Primary Sense (Gold Coast PHN), My Health Record (Australian Government), The Canning Tool (Arche Health Ltd) and manual collection (e.g. surveys or audits). (Note: cdmNET [Precedence Health Care] is another used primary care data extraction tool used in Australia, but it was not mentioned by study informants).

### Data linkage

**Survey questions: (Custodians) ‘*Has your dataset been linked with other datasets?…What other datasets have you linked to? Please list and also explain what methods/tools were used for data linkage’;* (Users) ‘*Have you linked any of the general practice/primary care datasets you’ve used with other datasets?… What methods or tools for data linkage did you use?’*** Eleven data custodians and 7 data users responded ‘Yes’ to data linkage, 1 custodian was ‘Not sure’ and a further 4 stated intention to link data. Most commonly linkage was with MBS or PBS data, or, for example, the Victorian Comprehensive Cancer Centre (VCCC) dataset which linked general practice EMR data (from NPS MedicineInsight [46] and the University of Melbourne’s Patron primary care data repository [47]), cancer registry, death index, hospital and administrative datasets within BioGrid Australia [48, 49]. Data linkage was referred to in Western Australia, but it was noted that primary care data were not readily available there. Data linkage was brokered via government and other organisations, e.g. the Western Australia Data Link, the Centre for Victorian Data Linkage, BioGrid Australia, the Centre for Health Record Linkage (CHeReL) and the Australian Institute for Health and Welfare (AIHW).

**Survey questions: (Custodians) ‘*Have you found limitations to the tool or methods used for data linkage? Please explain’;* (Users) ‘*If you encountered limitations related to data linkage tools or method(s) you have used, please describe them’.*** No survey respondents or interviewees suggested that data linkage ought to be avoided, numerous (n = 16) suggestions were made that linkage could increase utility or benefit of secondary use of primary care data in some way [37]; however, barriers to such linkage were many. Barriers related to data custodians not wanting to share data; the time taken to gain access to data and determine cost of access; technical issues (especially lack of consistent data linkage keys or IDs to facilitate linkage); and lack of governance, capacity, knowledge and trust. Some participants sought linkage of identifiable data to improve care of individuals. Data linkage was discussed in terms of linking disparate sets of health data or in terms of cross-sectoral data linkage (e.g. linking health data with justice, housing, social services, etc.) [37]. Table 3 provides more in-depth perspectives of interviewees on benefits and limitations of data linkage.

**Table 3:** Exemplar issues raised by interview participants on data linkage

**Benefits of data linkage for viewing patients’ journeys through the healthcare or cross sectoral systems**
*“We need better data linkage. Trying to have a look at what happens to an older person through the system, it’s improving, but it’s very difficult. How many services they access, hospital admissions, transition to nursing home, looking at predictors of those things. A whole lot could be done for people if we could build up profiles of risk factors, and that would be better for the (healthcare) system too. You can’t do that until you have a more complete dataset.” (Interviewee 7)*
*“I like data linkage… There are complex systems within multiple organisations, like health, justice, education; all these different systems that run separate things. I think it’s really important to understand a person’s journey through those different systems. I think the only way you’re going to do that is your data linkages… (It’s needed) to make decisions about policies around certain subjects, and how you deal with those populations… without that, they’re (policy-makers) just going blind.” (Interviewee 2)*
**Barrier: Lack of uniform approach and reticence to share data**
*“(Health data linkage) at a high level across the country would be ideal, because everyone is covered. But as it is now it's case by case and organisation by organisation, and it's all: ‘Do you want to share?’ And they say: ‘No’ or ‘Under these conditions’ so it's an ongoing battle to get the information you need.” (Interviewee 6)*
**Barrier: High time and cost to access linked data**
*“Each tranche of data to link is about $10,000 (Australian dollars)… I think it took about eight months to get data I requested, which is not too bad considering the stories I’ve heard… My PhD student waited three years for data on immunisation at post-code level from the Health Department.” (Interviewee 1)*
**Barrier: Insufficient departmental resourcing and knowledge limiting use of government data repositories**
*“I think a big limitation (of data linkage) is data sharing within the state (government)… (It’s) a really tough thing to do… But the issue is that Data Linkage (i.e. government Centre for Data Linkage) is such a small department for such a massive need. The other issue is… they expect that the requester has the ability to analyse that data. I would say less than 1% of DHHS (state government Department of Health) people have that skill. So, while I think data linkage is good and it’s a really valuable tool, it’s not really designed, currently, to allow a policy person or a manager, at the government level, to be able to use that data meaningfully.” (Interviewee 2)*

Quotes/table taken and adapted from [37]

### Cost of access

**Survey questions: (Custodians) ‘*Are people who access the data required to pay or provide something in return for access? Please describe what you get in exchange for sharing the data’.* (Users) ‘*Did you have to pay and/or provide something back to the data custodian in order to access the data? Please provide details about what you have in return for access’.*** Twenty percent (n = 6) of data custodians requested payment for data access. Half (50%, n = 16) of data users reported paying a fee (n = 11) and/or providing a service (such as recruiting general practices to share data with a custodian organisation) or supplying results of data analysis (such as reports to general practices who share data) in return for data access (n = 5). Some custodians also required co-author status on ensuing publications.

Of the 16 PHN-related data custodians, 12 did not receive any payment from those who accessed their data, but few reported allowing anyone outside of their organisation to access their datasets. Among *non*-PHN custodians, access fees varied, often “depending on the complexity of the data request, the type of data recipient (student, academic, commercial, government) and number of years of data provided” [37]. Fees expected or paid were not reported by most respondents, but those reported “ranged from no fee, $80 flat fee, $100 per randomised participant to $16,000, $20,000 or $30,000 (per data tranche)” [37]. A PHN responder raised the limitation of “increasing vendor costs of data extraction tools… (and that) enhancements and extended tool applications (to increase the utility of their data holdings) are costly” [37].

### Data quality frameworks

**Survey question: (Custodians) ‘*Are there any data quality frameworks or tools in place for (any of) the dataset(s)? Please describe the data quality framework(s) or tools’.*** A third of Data Custodians (n = 10) did not, or were not sure, whether they applied data quality tools or frameworks to their data. Of the remainder who did, various tools and processes were described including: workflow fixes to improve data input quality, use of benchmarking and data quality reports, management systems, guidelines, and privacy policies. Most ‘tools’ were bespoke and unpublished. Data quality frameworks named by participants included: *ABS Data Quality Framework* [50], *CSIRO’s Data61 De-identification Decision Making Framework* [51], a ‘*Department of Health Data Governance Framework’* and adherence to the *National Health Act 1953* [52].

**Survey question: (Custodians) ‘*Have you found any notable limitations of the data quality framework(s) or tools?’*** Eight (42.1%) reported ‘yes’, six (31.6%) that there were none, and one was not sure (four did not respond)” [37]. The **“notable limitations”** referred to by data custodians (Table 4), highlight variable conceptualisations of Data Quality Frameworks [37].

**Table 4:** Notable limitations of data quality frameworks

Lack of accessible and agreed standards: No agreed standard data quality framework (that is straightforward to apply) and no defined data coding/mapping standard
Shortfalls of “SNOMED Clinical Terms” in practical applications
Lack of resourcing and activities to support primary care providers to implement data quality improvements at point of data capture
The ‘resource drain’ for researchers or data custodians to implement a comprehensive data quality framework
Inconsistencies or lack of transparency around data transformation related to data extraction tools, leading to data quality issues including inconsistent or inaccurate results
Uncertainty among data custodians on types and definitions of data ‘de-identification’, leading to the possibility of secondary users re-identifying individuals in datasets
Technical limitations of received data structures and data tools limiting data recipients’ ability to analyse and report received data

Adapted from [37]

### Benefits and limitations

**Survey question: (All) ‘*What do you think are the benefits of secondary use of primary care datasets?’*** Perceived potential benefits of better use of primary care data were listed by most survey participants (n = 53) who collectively outlined benefits for individuals, populations, care providers, clinicians and staff, and for data end-users including researchers, data analysts and policymakers. Benefits included enabling more useful and higher quality research; and better evidence for service planning and policymaking to enable improvements to services, provision of care and health outcomes. It was suggested that comprehensive, longitudinal, raw ‘warts and all’ data could yield higher quality outputs “than a nicely curated or self-reported dataset where you can’t see what is missing” [37]. Benefits cited included using primary care data for trend measurement; monitoring/surveillance of disease outbreaks, drug safety and use of medicines; risk prediction and management; needs assessment at population and local levels; and evaluation of service and intervention quality, effectiveness of health policy and health care delivery. When referring to benefits of better use of primary care data, respondents frequently mentioned the role of data linkage in maximising the utility and meaningfulness of primary care-related data. Table 5 summarises participant perspectives on the limitations, benefits, barriers and enablers of secondary use of primary care data.

**Table 5:** Data custodian and user perspectives on benefits and limitations of secondary use of primary care data

	Synopsis of survey respondents’ perspectives on secondary use of primary care data (n = 53)
**BENEFITS (themes arising)**	
Intrinsic benefits of primary care data	Unique, rich, granular, ‘real world’ data with capacity to provide more population health information than any other health data source. Makes regional and remote-level information more accessible. Minimises measurement bias in research. When linked it creates systems view and triangulation, creating a ‘patient centred view’ of care pathways, patient needs and service gaps.
Assists policy and planning for provision of improved health services and health outcomes	Its analysis enables greater knowledge to assess and improve services in localities or broadly, service quality improvement (through competitive benchmarking), understanding of treatment outcomes and population health improvements. Provides an evidence-base for investment, interventions and efficiencies in health spending and technical infrastructure. Can inform policy and workforce planning. Contributes to a ‘Learning Healthcare System’.
Pragmatic research efficiencies & improvements	Data driven research can be rapid and cost effective leading to cost reductions. Big data (from large electronic medical record repositories) increases statistical power and increases research scale. Big data research can illuminate aspects of primary care otherwise not seen and can generate new research questions.
Patient generated data	Technologies can facilitate patient reported data collection through add-on apps, with potential to enhance primary care data.
Practice level	Enables providers to review their activities and make business improvements and ability to track and improve patient outcomes.
**LIMITATIONS (themes arising)**	
Technical and data capture limitations	Limitations from using or merging data from **non-standardised** clinical software systems and non-standardised data capture systems. Complex general practice workflows negatively impacting data quality. Clinicians who use paper-based rather than digital records.
Poor data quality, reliability	Data captured in non-standardised clinical software systems and extracted using non-standardised data capture systems leading to data inaccuracies and loss of data context. Poor use of existing field coding by clinicians and high use of free text limiting data utility (and adding to burden of data cleaning). Incomplete data fields (fields not captured by clinician or not extracted for secondary use). Difference between terms used by GPs and available SNOMED Clinical Terms prompting free text entries, and preferred terms changing over time, limiting data standardisation.
Data governance and access requirements limiting its usability	Requirements that data be de-identified or aggregated limits its utility. Lack of a minimum primary care/general practice data set in Australia containing data from all providers. Lack of a nationally endorsed patient unique identifier limits data linkage and identification of patient duplication. Limited permissions on what data can be linked and the ‘arduous’ nature of permissions process and cost to access data. Little incentive for primary care providers to share data leading to limited data available for secondary use.
Poor understanding of data complexity and context	Many data end-users unable to appropriately interpret the data because they do not understand its social and clinical context. Data collected for one purpose (clinical care) being used for purposes other than the primary purpose.
Unequal data representativeness	Lack of available data on priority populations including culturally and linguistically diverse, aboriginal communities, under-representation of vulnerable groups and over-representation of the ‘worried well’.
Privacy concerns, trust and ownership	Lack of community consultation on data use. Concern that shared data are stored off-shore or its use cannot be controlled. Unclear consent mechanisms and privacy concerns not addressed limiting clinicians sharing data. Varying ideas of who owns the data limiting the extent to which it is shared.
Lack of guidelines, policies, standards and ‘common data model’	The following limiting availability and utility of secondary primary care data use, lack of: national standards for general practice data quality and evaluation, clinical data capture system interoperability (too many clinical data systems), standardised data extraction tools, standard coding and common terminology; leadership to improve data standards and a ‘common data model’.

Adapted from [37]

**Survey question: (All) ‘*What do you think are the limitations of secondary use of primary care datasets?’*** Poor data quality was the most widely cited limitation of secondary use of primary care datasets. Poor quality necessitated expensive data cleaning and potentially unreliable outputs. Quality related limitations were said to arise from: clinician data capture (missing data, coding errors, extensive use of free text); design of clinical software systems (that varied in their intuitiveness and ease of data capture); GP workflow priorities and constraints precipitated by the design of the healthcare system (included data capture for clinical care not consistent with data capture required for research and service planning, and limited funding or incentive to change this); lack of standardisation of tools used to extract data from the clinical software systems; and the inherent complexity of pooling data extracted from clinical software systems that are not standardised nor interoperable. GPs’ deliberate omission of ‘sensitive’, stigma-associated diagnoses from EMRs, such as dementia and mental illness, was highlighted as a cause of inaccurate or poor data quality. One GP reported, in the lead up to opt-out participation in My Health Record, “deleting things (diagnoses in the EMR) all over the place that patients don’t want anyone to know about”.

Other limitations of secondary use of primary care datasets included: data analysts not understanding the social and clinical context of data, thus leading to incorrect data interpretation and therefore unreliable research, audit or surveillance outputs; primary care data lacking usability through absence of a unique patient identifier; lack of access to data; privacy and ownership concerns; data not adequately representing minority groups; lack of guidelines, policies and standards; and lack of a ‘common data model’ [37].

### Barriers and enablers

**Survey question: (All) ‘*What are the barriers to better use of primary care data?’*** The main barriers to better use of primary care data were described succinctly by one participant: “privacy concerns followed by technology and then data quality”. Barriers suggested by participants are outlined in Table 6, they related to fear (much of which was associated with lack of trust); leadership, governance and ethical constraints; lack of data availability; lack of access due to high cost or lack of awareness of dataset existence; lack of expertise and incentive; data linkage issues; technical issues; and health system and resource barriers. Lack of leadership and funding for ‘capacity building’ was described by a participant in terms of creating barriers to training, good process and generating expertise and incentive:

“(There is) lack of GP leadership, a weak academic GP sector with almost no funding of capacity building in the sector; many skill sets are needed including in medical informatics and statistics. Research funding opportunities and success rates are poor for large projects in primary care. To do GP research we need motivation, funds and an easy way of doing it. Primary care data provides a relatively cheap and easier way to obtain significant research outcomes but funding to build the datasets and the trained people to undertake the work needs boosting.” (Interviewee 3) [37].

As outlined in Table 6, some perceived barriers to secondary use of primary care data were associated with fear or lack of trust among stakeholders. For example, barriers to data sharing and linkage including: politician’s fear of reputational damage diminishing government support for and facilitation of data linkage: “It’s not about risk of a breach, it’s about risk of embarrassing a politician… they want zero risk”; “fear that the data could be used against the GPs…(and) used in a way that is out of context and incorrect” (i.e. lack of trust in how the data will be used) leading to GP refusal to grant access to their EMR data; “fear of data breaches” leading to unwillingness to share (lack of trust of data security measures of others); and “fear… that people (GPs) don’t believe that the quality of data is good enough to share, they’re a bit afraid that what they’re sharing might be wrong” [37].

**Table 6:** Data custodian and user perspectives on barriers and enablers of secondary use of primary care data

	Synopsis of survey respondents’ perspectives on secondary use of primary care data (n = 53)
**BARRIERS (themes arising)**	
Fear, reticence and lack of trust	GP concerns for patient **privacy** and not perceiving value in secondary data use impacting willingness share data. Fear or **lack of trust of data security**. Fear of privacy breaches or ‘illegal’ data use resulting in harm. GPs’ fear that they record data of insufficient quality for sharing. Fear that sharing data may increase government control of GPs (lack of trust of government).
Leadership, governance & ethical constraints	**Leadership, legal and regulatory issues:** confused determination of who ‘owns’ or is ‘in charge’ of data. Federal-state divide and no ‘national approach’ to data collection. Limited engagement between key stakeholders. Protection of intellectual or commercial interesting as barrier to coordination of effort to optimise data use, thus leading to duplication of effort. **Ethics and governance:** Barriers to access including stringent ethical constraints, data governance protocols, data access controls and confidentiality restrictions. Lack of transparency of consent models, governance processes and methodologies leading to lack of trust in data sharing. Expensive, cumbersome and slow processes for data access approvals. Lack of clarity on what constitutes ‘deidentified’ data and concern about sharing ‘deidentified’ data without explicit patient consent.
Lack of data availability	Lack of available longitudinal patient data. Incomplete data entry by service providers.
Lack of access due to cost or awareness	Limited knowledge about what data are available and how to access it. Prohibitive cost to access data for research. The high costs charged by vendors to use their data extraction tools and to access extended tool applications and enhancements.
Lack of expertise, experience & incentive	Too few clinicians involved in planning data analyses and in reaching research conclusions. General practice staff not motivated to collect clean, accurate and complete data. Absence of shared vision/capacity to build systems to utilise current non-standardised data sources.
Barriers to data linkage	Inability to link patient data. Lack of a reliable individual person identification numbers for data linkage. Lack of availability of, and access to, some datasets needed for linkage (lack of stakeholder agreement and governance arrangements).
Technical systems barriers *(and lack of systems to improve data quality and quantity)*	**Lack of standardisation and interoperability** of electronic medical records (EMRs) and their coding, classifications, data definitions, and of data extracted using different extraction tools, leading to variable data structures and quality decreasing data utility. Inconsistent and poor mapping of medical terms within clinical software systems. Data extractions tools unable to collect from all clinical software systems. Poor data quality (completeness, cleanliness, granularity) as barrier to better use. Inadequate national digital health record, lack of primary care minimum data set and lack of consensus on what a minimum dataset should include. Relatively few providers of data warehousing.
Health system & resource barriers	**Structural:** Most primary care providers as private businesses where owner has choice in data capture systems and voluntary data sharing; and the public being free to visit any practitioner, a barrier to longitudinal patient records. **Workforce:** The high general practice staff turnover negatively impacting data input and quality (brain drain). **Timelines:** slow release of data affecting timeliness of evaluation and needs assessments. **Funding/Cost:** Lack of funding to collect and analyse data and to support the implementation of findings; Lack of motivation, capacity, resources and education to prioritise data input and improve data quality; Lack of research resources to interpret data (including for Primary Health Networks to interpret for planning purposes); Insufficient research and skills/capacity building funding for the academic primary care sector.
**ENABLERS (themes arising)**	
Qualities	Build primary care provider and public **trust** of data custodians and users. **Reassurance** of appropriate use of data. Grow **awareness** and **knowledge** of the **value** and application of primary care data (create shared vision). Ensure **transparency** in data access and use. Use **innovative** and forward-thinking solutions. **Altruism** prompts data sharing.
Leadership	**Leadership from**: **Universities** (for expertise and engagement in secondary use); **Primary Health Networks** (utilising their relationships with general practice); **GP Colleges**; and other organisations as appropriate
Governance	Improved, ‘tighter’ or ‘clearer’ governance with: unambiguous and agreed strategic framework(s), **agreed processes,** clarity of government position, incorporation of **robust safeguards** for dataaccess and use. Clearer guidelines and steps on how to **deidentify data** and recognise when data are no longer deidentified and complies with both state and national privacy and data protection legislations. Support for **national adoption of a single GP data extraction tool** and **centralised coordination** and management of GP data (to decrease duplication of effort). Incentive payments to clinicians to encourage improvement of data quality & data sharing
Partnerships and capacity building	Facilitate **engagement** between key stakeholders: clinicians, consumers, government, researchers, Primary Health Networks. Expand practice-based research-oriented networks to facilitate access to primary care data. **Educate:** Open pathways to greater secondary use of data by turning clinicians’ data into knowledge delivered back to them for business and care improvement. Have clinicians benefit from review and audit of their own data so they experience how accurate vs inaccurate data capture can benefit or limit them. Build researchers’ capacities to access and interpret data. Enable consumers to understand the research value of primary care data (especially when linked to other datasets) and build on public expectation/perception that policymakers may already use linked data systems to improve services. **Raise cross-sector capacity** of and willingness for data linkage.
Technologies and method development	Better use of secondary data through advancement in computing hardware and software technologies, **interoperability** of data collection tools, or adoption of a single extraction too capable of working across multiple vendor software packages. Improved portals for practice display of data to encourage continuous quality improvement in data capture. Advancement in: data storage and IT security, technical cross-sectoral capability to enable data linkage, **data and coding standardisation**/consistency, systematic data quality assurance, mechanisms for appropriate data interpretation.
Resources	**Funding to:** Train primary care staff and clinicians in health informatics and educate on data value and best practice data collection, quality improvement and better use of own data (**workforce upskilling**). Have computer scientists and health informaticians support practices to capture quality data and enable its use. Build a national primary care dataset that is accessible and affordable for researchers and provide incentives to primary care practices to share data. **Extraction tools** that meet data user-needs. **Time** to demonstrate good outcomes resulting from secondary use of primary care data.

Adapted from [37]

Lack of clarity around patient consent and deidentification protocols were also described as significant barriers. An interviewee referred to discrepancies between guidelines from the Office of the Information Commissioner [53] and the RACGP [54], saying: “people aren’t clear when you need consent and when you don’t”, nor were they clear about what constitutes truly deidentified data. A key barrier for researchers included lack of transparency around consent models and governance; they reported needing “to negotiate cumbersome, slow, expensive processes for gaining ethics and Data Custodian approvals to access data” [37].

**Survey question: (All) ‘*What are the enablers to better use of primary care data?’*** Many enablers suggested by researcher respondents (Table 6) centred on improving access to data through making it more affordable and streamlining processes for gaining access. In contrast, enablers suggested by PHN respondents tended to be around developing business models, moving away from the restrictive format of data delivered to PHNs by third party providers, and building trust and educating GPs and non-clinical staff on the benefit of data-driven quality improvement. Researchers tended to be less concerned about ‘poor quality’ data and data format than PHN staff were, and argued that longitudinal ‘warts and all’ data were of higher quality than curated (transformed) or self-reported datasets.

System and data transparency, data dictionaries documenting data transformations, and GP/clinician advisors or clinician-researchers, were considered key to ensuring that quality outputs were possible even when research was based on ‘dirty’ data. A participant quote exemplifies ideas expressed about how to improve use of primary care data and quality of outputs:

“When analysing data for research purposes, whether the data were collected specifically for a particular study or came from secondary sources, the analyst needs to understand why, how and when the data was collected, as well as how it was processed, including the coding, cleaning and formatting of the data as all these can potentially introduce biases. There should be well developed and transparent standard operating procedures (SOPs) for all these data collection steps, with input from a range of individuals involved in collecting, preparing and analysing the primary care data, including the clinicians right through to the data analysts/statisticians/end point users. Individuals across the data collection continuum should be trained on these SOPs to ensure data quality and consistency in coding and data cleaning processing. There should be continual communication between all the individuals across the continuum from when the data are first collected to the endpoint when analysed and outcomes reported. The analyst can also give feedback on how data and data quality can be improved to enable better use of primary care data.” (Survey respondent) [37].

#### Building data use capacity

**Survey question: (All) ‘*How do you think that primary care datasets could be better used to support improve health outcomes?’*** We categorised the open answer suggestions on how primary care data could be better used into the following themes: “research solutions; data linkage; technical data-led solutions; surveillance and monitoring; inform/support general practice; health promotion solutions; and policy, governance and system changes” [37]. Suggested strategies for better use of primary care data lacked consensus across different types of participants; however, they included: centralised coordination and management of GP data; national use of both a single GP clinical software data collection tool and a single data extraction tool; removing the voluntary nature of GP data sharing; and “building on existing public expectation that policy-makers are already using linked data systems to improve services” [37].

Technical solutions to support data linkage, adoptions of data standards, and use of unique person identifiers, were broadly supported. Some participants focused on the need to give more support at the general practice provider level to increase, among those capturing data, understanding of why data quality is important. An interviewee outlined their ‘vision’ for improving use of primary care data, much of which aligned with the views of other participants:

“My vision is about building the infrastructure and then the source of questions that could be answered is multitudinous. So, health data that is collected and coded correctly, improving the software so that it makes it easy, linking it, being able to follow the patient journey, having absolute best practice in terms of security, and then having a really good process for ensuring that sensible questions, that are answerable, are asked of the data with an adequately skilled workforce to do that. That could produce very powerful outcomes in terms of the community getting to know itself better, understanding its issues better, being part of the conversation, which we need to have about bringing everybody along with the hope to improve health outcomes. Health outcomes writ large. It's not about just health. It's about linking with education, linking with correctional services, linking with housing, linking with all of those databases needs to happen. And that isn’t a conversation that seems to be happening, it's all just about the health databases at this stage. So, take Scotland as our example and move on.” (Interviewee 3) [37].

## Discussion

This was the first comprehensive study to identify primary health care-related datasets used for secondary purposes in Australia, to gain insights into the availability and nature of these datasets and their data quality frameworks, and to understand data user and data custodian perspectives on what is needed to build capacity for better use of primary care data for secondary purposes. Participants nominated more than one hundred primary care-related datasets, developed for many different purposes, often not assessed within a quality framework, and with significant barriers to their secondary use, including concerns about data quality, access, expertise, consent and cost. A perceived fundamental barrier was clinicians’ concern that sharing EMR data increases risk to them related to breaches of patient privacy and data security, with the perceived safe option being to deny access for secondary use.

Based on the perspectives of the participants, which align with the views of the Australian Productivity Commission [3], there are many opportunities and benefits associated with better use of primary care data, including benefits arising from data linkage. Adoption of measures leading to realisation of benefits requires widespread buy-in and also agreement from those stakeholders whom may have commercial, intellectual property or other reasons to object to a more standardised approach to data collection (e.g. owners of bespoke data capture software, data collection tools, systems or datasets), or mandated adoption of measures.

Our identification of the wide array of primary care-related datasets—which included health workforce, hospital admissions and Australian Bureau of Statistics data suggests (1) that there is broad conceptualisation of what a primary care dataset is and similarly broad conceptualisation of ‘secondary use’; and (2) that there is likely to be duplication of activities related to general practice EMR or other patient-related data collection, governance, cleaning, analysis and interpretation. In 2008 the Australian Institute of Health and Welfare found limited data available to build a comprehensive picture of general practice, what they found was of patchy quality [4]. Ten years later we found that primary care data was still difficult to access for secondary use such as health planning, but there was more of it and there are plans to do more with it, such as the developing national minimum primary care data asset [6, 27, 30].

Australia’s multitudinous repositories of primary care-related data used for reasons other than clinical care, captured using different commercial clinical software systems that use different coding classifications, collected via different data extraction tools, for different purposes [28], and with extracted data cleaned and mapped to different classificatory systems, results in non-standardised datasets being analysed giving rise to different denominators, potentially leading to different conclusions based on inquiry into the same question [55]—this is indeed a problem for data-driven healthcare improvement.

### Data quality frameworks

A third of data custodians did not, or were not sure whether they used a data quality framework, this suggests some may have been unfamiliar with the terminology while others did not have processes for checking data quality. Among those who did report using one there was no common conceptualisation of what a data quality framework was. The breadth of datasets noted in this study may be too broad for application of a single, standardised data quality framework, but our findings suggest that, at least in Australia, more needs to be done to make available to data custodians information on implementation of frameworks for data and metadata quality assurance.

Data quality frameworks are garnering increased attention to ‘harmonise’ data quality and its assessment to establish a common understanding of the strengths and limitations of EMR data [38, 56, 57]. Such frameworks require consistent use of common terminology. In the health arena there is no standard data quality framework. Kahn’s *Harmonized Data Quality Framework* [56] is being applied in Australia through the AHRA Transformational Data Collaboration and the Australian Institute for Health and Welfare [38]. Adoption of appropriate data quality frameworks enables rigorous documentation and assessment of metadata and data quality for meaningful, contextual use of the information contained in a dataset [37].

### Data standardisation

Lack of data standardisation and interoperability of clinical data software and extraction tools, barriers raised by participants and commonly noted in Australian and international literature [14, 19, 28, 58], are remedied by adoption of a unified interoperability framework [59] or standardised software collection and/or data extraction tools; barriers to the solutions, however, are many. Respondents’ governance-related solutions focused on centralisation of datasets for unambiguous application of quality frameworks, data structures and coding standards, security safeguards, and to de-identify data in a consistent manner meeting clear legal and ethical standards. The adoption of a single, centralised dataset was not, however, universally sought, for example, because of threat to competition and acknowledgement that competition between vendors and data custodians “drives change and innovation” [60]. In the UK and US, vendor competition is maintained but only accredited or certified EMR vendor systems can be adopted by the UK’s National Health System (NHS) providers [34], and by US Medicare providers if they are to receive full reimbursement via quality payment programs [61]. Accreditation/certification ensures that EMRs meet functional requirements including core sets of clinical data elements in pursuit of interoperability and excellence in health information and technology [34, 61, 62]; but there are no signs of similar accreditation measures being taken in Australia. Legal frameworks and social licence are also needed for centralised data collection. In Denmark, legal review led to the 2014 demise of the centralised Danish General Practice Database when it was determined that its general clinical data content did not meet Denmark’s legal definition of a clinical database (i.e. a disease specific register) [15], and lack of social licence led to the closure of the UK’s care.data program [63] and 2021 stalling of NHS Digital’s General Practice Data for Planning and Research program’s data collection launch [64].

As well as lacking standardisation, the complex data structures within different vendor EMR software systems in Australia mean that raw data are not well structured for research use. The application of common data models (CDMs) or open-source data quality tools such as from OMOP (Observational Medical Outcomes Partnership [65]), openEHR or others [62, 66], is said to support ‘interoperable knowledge representation’ which will increase availability of high-quality clinical data for research [62]. Some Australian vendors in Australia are working to integrate HL7 standards [67] and FHIR (Fast Healthcare Interoperability Resources) [68] into point of care software systems; however, applying external standards to systems not designed to conform can be complicated and leaves room for error, especially if the standard incorporates a large array of specifications. For research data use, OMOP CDM uses a simplified set of 17 tables and while it does not provide a structure for point of care data collection, as a simplified open-source tool it can fast-track data transformations and sharing of learning through enabling researchers to more quickly understand data, review trends and make data comparable across systems within and between countries [62, 65]. Use of raw data and data transformed to conform to complex or simplified CDMs or exchange standards, have their place, and there is balance to yet be found between maintaining flexibility within point of care data collection tools versus forcing clinicians to code their data entries.

### Making the most of real-world data

As described by participants, patient care is a GPs’ priority and so capture of data in any way beyond that needed to meet that priority is secondary. GPs tend to code little of their data [55, 69] and there is evidence that financial incentives to code data can diminish the person-centredness of consultations and prompt data capture gaming that leads to further data inaccuracies [70]. An Australian study of GP attitudes found half of those interviewed were concerned that their inaccurate recording of information in EMRs, due primarily to time constraints, could cause key data to be missed and therefore incorrect interpretation of data used by researchers, this was therefore a reason they preferred not to share clinical data for secondary use [71]. To improve data completeness and quality at source, system changes are required to enhance the ease of clinical coding, to allow sufficient time for data capture and engagement and partnership with GPs to demonstrate the importance and benefit of maintaining data quality [72]. Nonetheless, through understanding the raw data and any transformations made to it, researchers and health data informaticians can work successfully with poorly coded data. Understanding the limitations and context of data capture are key, as lack of analyst awareness of dataset bias can lead to inappropriate outputs from secondary use of primary care data which were considered by respondents to potentially undermine general practice and public support of such data being shared. This reiterates the importance of retaining metadata or other information about data sources and transformations and so preserve data integrity [12, 73]. Phenotype algorithm development is also progressing to better characterise patients from both coded and free text data [74, 75].

### Building trust

Fear-related themes were frequently highlighted as preventing “better use of primary care data” (including data for linkage). Developing trust to allay fear reinforced many of the “enablers of better use of primary care data” suggested by participants. Public (dis)trust has long been discussed with regard to secondary use of health data, for the public [16, 76–78] and care providers [2, 22, 79]. Building trust, as suggested by respondents, requires strong and transparent leadership and governance, transparency of end-to-end data-related processes, “meaningful stakeholder engagement, shared vision, robust data security and privacy protection, and publicised outcomes to demonstrate benefit and provide reassurance that data custodians and end-users are ‘doing the right thing’” [37]. Adoption of standardised and/or interoperable data collection, coding and data quality assurance tools would lead to process efficiencies and building trust between clinicians, third party data custodians, the public and data end-users should improve data quality and efficiencies related its secondary use. These expectations fit with attitudes determined elsewhere [2, 77]. Participant stakeholders also called for greater leadership from GP colleges, universities, PHNs and data custodians, to provide strong and transparent governance frameworks.

### Strengths and limitations

It is a strength of qualitative research that stakeholder perceptions can be made explicit, and we have reported these in context of the questions from which they arose. Perceptions include subjective value judgements, some of which may be considered misconceptions. It is beyond the scope of this paper, however, to critique each opinion for its value, but we do note that if misconceptions persist and become widespread, they have the potential to become barriers to moving forward. For example, we report the perceived limitation that “requirements that data be de-identified or aggregated limits its utility”, whereas there are innumerable examples in Australia and internationally where, for secondary purposes, privacy protecting, de-identified or aggregated data have enormous utility [2, 48, 53, 80].

The purposeful and snowball sampling approach ensured that known and possible users and custodians of primary care data were invited to participate; however, their self-selection and identification as secondary ‘users’ or ‘custodians’ of datasets used for secondary purposes, coupled with differing understandings of “secondary use”, may have prevented some potential participant from completing the survey even though we may have considered them custodians or users. This limits the completeness and generalisability of the findings. Seventeen participants marked themselves as ‘ineligible’ at the first question (thereby exiting the survey). A further 58 persons were not included due to insufficient survey completion.

Another limitation was that some participants, either to protect their anonymity, commercial or research interests, or through lack of engagement with the survey, gave vague descriptions of the datasets they manage, use or know. This resulted in many gaps in the list of named datasets and limited our ability to ‘map’ available primary care datasets as thoroughly as initially planned [37].

Data collection ceased in November 2018; this means that changes since then in the Australian primary care data environment are not reflected. The QI PIP was introduced in August 2019 [33], consultation around developing a National Primary Health Care Data Asset has since commenced [32], and ‘Lumos’, a NSW Health Department primary care data linkage initiative has commenced (https://www.health.nsw.gov.au/lumos) [37].

### Next steps

In Australia there is little sign that commercial EMR vendors servicing primary care will adopt a single, underlying data structure (and this may not be desirable). Conformance work to FHIR data interchange standards and developing enhanced means of clinical coding data is underway however without overarching system-wide accreditation processes, conformance and wider systems compliance is variable and subject to funding priorities; multiple strategies are then needed to ensure best use of real world-data while striving to achieve improved data capture at point of care. Pragmatism is required and at this stage, promoting the transformation of the many data structures underlying the many EMR systems to common data models and addressing the many process duplication, data quality, data transformation and data interpretation issues is important.

The HPSR approach of this study incorporated stakeholder feedback of preliminary findings which led AHRA representatives to conclude that before taking steps to seek government leadership on a national approach to data standards and linkage, more immediate issues to be addressed were supporting increased health data useability and thereby building both data and data-user capacity. This led, in 2019, to the creation of AHRA’s Transformational Data Collaboration (TDC) and to work with a wide range of partners to: curate and develop medical terminology mapping and phenotypes; establish a national, research ready hospital EMR data asset utilising the OMOP CDM; convert the Patron primary care dataset to OMOP CDM; develop a new data quality tool (White Bandicoot) that can run quality metrics on EMR data and visually highlight quality issues; establish OHDSI Australia (Observational Health Data Sciences and Informatics OHDSI to provide no cost training to CDMs; and to develop FHIR terminology services to be compatible within OMOP tooling [81]. The outputs of these project will lead to new pathways for research and health data use to further inform policy and infrastructure development.

A national approach should seek to incorporate harmonisation of or guidance around differing state-based legislation that affects use of health data, and government leadership on accreditation of EMR clinical software systems so that Australian systems must adopt agreed data and quality standards as is done elsewhere [34, 61].

## Conclusion

There are many primary care-related datasets in Australia used for purposes other than clinical care; their large number suggests duplication of labour related to data collection, preparation and utilisation. ‘Better use’ requires technical, process and governance solutions to address limitations and improve data quality. While benefits of secondary use are many, finding ways to reach consensus on how to address limitations and barriers requires strong leadership, especially for adoption of data and quality standards and of phenotype algorithms for standardising condition definitions. A national approach might seek to incorporate harmonisation of or guidance around differing state-based legislation that affects use of health data. The principles of transparency, partnership and security should guide the collective drive to better use of primary care data to improve health outcomes.

## Supplementary Information

**Additional file 1:** Copy of online survey: Main data collection tool (with branching logic that enabled separation of some questions for data custodians and data users).

**Additional file 2:** Interview question/theme guide.

## Data Availability

The dataset supporting the conclusions of this article is available from the corresponding author on reasonable request.

## Abbreviations

ABS: Australian Bureau of Statistics
AIHW: Australian Institute of Health and Welfare
CDM: Common Data Model
CSIRO: Commonwealth Scientific and Industrial Research Organisation
EMR: Electronic Medical Record
ePBRN: Electronic (data-based) Practice Based Research Network
GP: General (medical) Practitioner—i.e. a Family Physician
HPSR: Health Policy and Systems Research
IP: Intellectual Property
MBS: Medicare Benefits Scheme (government funding of public health services)
PBS: Pharmaceutical Benefits Scheme (government medicines funding)
PHN: Primary Health Network (independent organisations funded by the Australian Government to increase efficiencies and effectiveness of health services for patients)
QI PIP: Quality Improvement Practice Incentive Payment
RACGP: Royal Australian College of General Practitioners
TDC: Transformational Data Collaborative
