# Supplementary material for: Identifying primary care datasets and perspectives on their secondary use: a survey of Australian data users and custodians
Source: BMC Med Inform Decis Mak. 2022 Apr 6;22:94. doi: 10.1186/s12911-022-01830-9 (PMC8988328; doi:10.1186/s12911-022-01830-9)
Supplement: Supplementary file 1 — Additional file 1: Copy of online survey: Main data collection tool (with branching logic that enabled separation of some questions for data custodians and data users). [file 12911_2022_1830_MOESM1_ESM.pdf]

Mapping Australian primary care datasets and building data use capacity

Please complete the survey below.

If you have any questions please email xxxxxx.xxxxxxx@unimelb.edu.au or phone Xxxxxx Xxxxxxx on (03) 8344 xxxx at the Department of General Practice, The University of Melbourne.

Thank you!

Welcome and consent

There is increasing interest in 'secondary' use of general practice data in Australia, but no one has a clear picture on what data is collected for secondary use, by whom, and what is being done with it.

The results of this survey will provide a clearer picture of primary care datasets in Australia (being used for secondary purposes).

This project is part of national capacity building work to prompt data-driven healthcare improvement. It is an initiative of AHRA (Australian Health Research Alliance) and MACH (Melbourne Academic Centre for Health), funded by the Medical Research Future Fund (MRFF).

Your completion of this survey will take around 5-15 minutes. This time variation is because many of the questions allow open ended responses (less or more detailed).

On completing and submitting this survey your consent to participate is implied.*

* By submitting the survey you acknowledge that you have read and understood the Consent and Participant Information and you freely agree to participate (see attached Participant Information pdf).

If you have any questions please contact Dr Xxxxxx Xxxxxxx at The University of Melbourne: (03) 8344 xxxx or xxxxxx.xxxxxxx@unimelb.edu.au

Findings will be discussed at the Primary Care Data for Health Care Improvement and Research workshop, 26 November 2018, Sydney Australia. www.nhmrc2018.com.au/pre-post-events.php

[Attachment: "Participant Information Statement_AHRA Survey.pdf"]

Please check the box if you would like to be named in acknowledgements of any published results as having contributed to this research:	◯ Yes, I would like to be named in acknowledgements (You will be named only if your survey is submitted as 'complete'.)
Please enter your name and affiliation:	__________________________________
Please check the box if you would like a summary of the results emailed to you at the conclusion of the project:	◯ Yes, please email me a summary of results
Please enter your email address to receive the summary of results:	__________________________________ (Any personal information you give is kept strictly confidential and is not passed on to any third parties.)

Eligibility check

Some electronic medical records from Australian general practice or other primary care services are used for secondary purposes including research, audit, surveillance, and quality improvement.<br><br>Have you used, or do you have responsibility for, a primary care dataset that is used for secondary purposes?	◯ Yes ◯ No

Data Custodian or Data User?

Do you consider yourself a Custodian or 'owner' of one or more general practice or other primary care datasets?	◯ Yes ◯ No<br>(A Data Custodian takes primary responsibility for dataset access, security, data integrity, etc.)

Questions for Data Custodians

What is the name of the dataset you are Custodian of or have responsibility for, and/or where is it located?

If you are responsible for more than one, please list all.

____

What state(s) and/or localities does the majority of the data come from?

____

Please describe characteristics and purpose of the dataset(s).

____
(* Be as brief or detailed as you like.)

Please describe any data governance and consent mechanisms that are in place around the dataset(s).

____

Are there any data quality frameworks or tools in place for (any of) the dataset(s)?

◯ Yes ◯ No ◯ I'm not sure

Please describe the data quality framework(s) or tools:

____

Please briefly describe why you are 'not sure' whether there are data quality frameworks or tools in place:

____

Have you found any notable limitations to the data quality frameworks or tools you have used?

◯ Yes ◯ No ◯ I'm not sure

Please briefly describe the limitations of the data quality framework(s) or tools:

____

Who can access the dataset and how?

____

Are people who access the data required to pay or provide something in return for access?

◯ Yes ◯ No ◯ I'm not sure
(For example a: fee; service; report or other information; etc.)

Please describe what you get in exchange for sharing the data:

____

Has your dataset been linked with other datasets?

◯ Yes ◯ No ◯ I'm not sure

What other datasets have you linked your dataset to? Please list and also explain what methods / tools were used for data linkage:

____

Have you found limitations to the tool or methods used for data linkage method(s)? If yes, please explain: __________________________________

Do you intend for your dataset to be linked with other datasets? ◯ Yes ◯ No ◯ I'm not sure

Secondary users of general practice and other primary care datasets

You have indicated that you are not a Data Custodian but you have used general practice or other primary care datasets for secondary purposes.

Please briefly describe the nature of your interaction with these datsets: ____________________

What primary care or general practice datasets have you used for secondary purposes? Please list them and describe how you gained access to them: ____________________

Did you have to pay and/or provide something back to the data custodian in order to access the data? ◯ Yes ◯ No ◯ I'm not sure

Please provide details about what you gave in return for access to the data: ____________________

Please describe what elements are important to you when accessing or using primary care / general practice datasets for secondary purposes: ____________________

Have you linked any of the general practice / primary care datasets you've used with other datasets? ◯ Yes ◯ No ◯ I'm not sure

What methods or tools for data linkage did you use? ____________________

If you encountered limitations related to data linkage tools or method(s) you have used, please describe them here: ____________________

Mapping primary care datasets

Please list other general practice or primary care datasets that you are aware of (you may or may not have used them):

____

(If you are not aware of any other primary care datasets being used for secondary purposes, please say so.)

Building capacity of primary care datasets - to support improved health outcomes

How do you think that primary care datasets could be better used to support improved health outcomes?

What do you think are the LIMITATIONS of secondary use of primary care datasets?

What do you think are the BENEFITS of secondary use of primary care datasets?

What are the BARRIERS to better use of primary care data?

What are the ENABLERS to better use of primary care data?

Do you think there is an ideal way to link primary care datasets? If yes, please describe it:

If you would like to share more thoughts on data / dataset quality, please do so here:

About you - and further comments

What type of organisation do you work for (related to your work with primary care data)?

- ☐ Educational institution
- ☐ General Practice
- ☐ Government
- ☐ PHN (Primary Health Network)
- ☐ Pharmaceutical
- ☐ Insurer
- ☐ Other

Please describe the type of 'other' organisation(s).

____

In what state or territory are you based?

◯ ACT ◯ New South Wales
◯ Northern Territory ◯ Queensland
◯ South Australia ◯ Tasmania
◯ Victoria ◯ Western Australia
◯ National ◯ Elsewhere

Please describe where 'Elsewhere' is:

____

About how many different Australian primary care datasets have you accessed for secondary purposes?

◯ None ◯ 1 ◯ 2 ◯ 3
◯ 4 ◯ 5 ◯ 6 ◯ 7
◯ 8 ◯ 9 ◯ 10 ◯ About 11 - 20
◯ About 21 - 30 ◯ About 41 - 50
◯ More than 50 ◯ More than 100

In what year (approximately) did you first access a primary care dataset for secondary use?

____

How did you hear about this survey?

◯ Direct email from the researchers
◯ Link was forwarded by a colleague
◯ RACGP
◯ Social Media
◯ Other
(Please choose the closest single option.)

If 'other', please describe:

____

If you have more to say about secondary use of primary care datasets and data linkage, please tell us here:

____

Would you like to be contacted to contribute further information via phone?

◯ Yes
◯ No

Your name:

If you do not want to leave your details you can phone Dr Xxxxxx Xxxxxxx on (03) 8344 xxxx, xxxx xxx xxx or email xxxxxx.xxxxxxx@unimelb.edu.au

____
(Any personal information you give is kept strictly confidential and is not passed on to any third parties.)

Phone number:

____
(Any personal information you give is kept strictly confidential and is not passed on to any third parties.)
