# Supplementary material for: Identifying primary care datasets and perspectives on their secondary use: a survey of Australian data users and custodians
Source: BMC Med Inform Decis Mak. 2022 Apr 6;22:94. doi: 10.1186/s12911-022-01830-9 (PMC8988328; doi:10.1186/s12911-022-01830-9)
Supplement: Supplementary file 2 — Additional file 2: Interview question/theme guide. [file 12911_2022_1830_MOESM2_ESM.docx]

Interview question theme guide

*This project aims to gain deeper understanding of availability and secondary use of routinely collected general practice data. If you agree to be interviewed, the questions asked will explore the following areas:*

1. Briefly: your professional role and the type of organisation you work for.
2. Whether you are a Data Custodian / Data Steward or a user of general practice data.
3. Identifying general practice datasets in Australia, including their:
   1. Purpose
   2. Governance and consent mechanisms
   3. Data quality frameworks
   4. Data linkage capability
4. What you consider to be the ideal way to link data – and barriers and facilitators of this?
5. What you consider to be the main benefits and limitations of secondary use of general practice datasets?
6. Ways that general practice datasets could be better used to support improved health outcomes – including barriers and enablers to better user.
7. How current systems of healthcare related data sharing could be improved to better prompt research and knowledge discovery?
8. Suggestions of other people in Australia that might be appropriate to be interviewed as part of this study.
